# Can big data reduce urban environmental pollution? Evidence from China’s digital technology experimental zone

**DOI:** 10.1371/journal.pone.0288429

**Published:** 2024-10-16

**Authors:** Jiahao Zhang, Fusheng Liang, Peng Gao

**Affiliations:** 1 College of Economics and Management, Shanghai Ocean University, Shanghai, China; 2 Institute of Food and Strategic Reserves, Nanjing University of Finance and Economics, Nanjing, China; 3 School of Management, Guangdong Ocean University, Zhanjiang, China; Huazhong University of Science and Technology, CHINA

## Abstract

This paper investigates the impact of the digital economy on urban environmental pollution by analyzing panel data from 283 prefecture-level cities in China from 2011 to 2019 and using the digital technology comprehensive pilot zone of China as a natural experiment. The results demonstrate that digital technology has a significant effect in reducing pollutant emissions and empowering urban environmental governance. The findings are proven to be robust based on various tests, including parallel trend, PSM-DID, and placebo tests. Our analysis further shows that digital technology is particularly effective in controlling pollution in old industrial areas, high digital areas, and low energy efficiency areas. We also find that the national digital technology integrated pilot zone can mitigate environmental pollution in prefecture-level cities by increasing public environmental awareness and encouraging green technology innovation. Moreover, our research indicates that digital technology-enabled urban pollution control can contribute to the formation of a new urbanization pattern in China. These findings provide valuable insights for promoting the digital economy and achieving the goal of carbon reduction in China.

## Introduction

In recent years, China’s economy has maintained rapid growth, but issues such as environmental pollution, resource scarcity, and ecological destruction have become increasingly challenging. "We need to coordinate efforts to reduce carbon emissions, combat pollution, expand green spaces, and promote growth, advance ecological priorities, advocate resource conservation, and green, low-carbon development." The 20th National Congress report of the Communist Party of China once again emphasized the importance of pursuing green development. The Chinese government has invested significant resources in green transformation, with the total investment in environmental governance reaching 1.06389 trillion yuan in 2020. Faced with the challenge of tackling environmental pollution emissions, local governments are attempting to break away from the traditional development model characterized by high pollution and high energy consumption and promote the transformation and upgrading of green, low-carbon industries. In this regard, achieving breakthroughs amidst the dual constraints of environmental resources has become an urgent issue in urban environmental governance.

In the digital age, the integration of traditional industries with digital information is driving the world economy toward a greener direction [1]. In the wave of big data that is sweeping the globe, China is actively embracing big data to improve its green development pattern and achieve governance breakthroughs. Empowered by digitalization, information and communication technology (ICT) is deeply integrated with various aspects of the economy and society, effectively alleviating the imbalanced and insufficient development pattern in China [2]. Big data, as a key production factor of the digital economy, supported by emerging network infrastructures such as blockchain and 5G, optimizes the aggregation of urban resources and stimulates green technological innovation, thereby helping reduce environmental pollution emissions [3]. In 2015, the "Action Plan for Promoting the Development of Big Data" was issued, explicitly calling for the "launch of regional pilot programs," marking the gradual exploration of big data demonstration zones in China. Nearly ten provinces (direct-controlled municipalities) such as Guizhou Province and Beijing(List of Big Data pilot zones: Guizhou, Beijing-Tianjin-Hebei, Pearl River Delta, Shanghai, Henan, Chongqing, Shenyang and Inner Mongolia. Data is from《The 14th Five-Year Plan of the National Big Data Comprehensive Pilot Zone》) Municipality have successively promoted the construction of national-level comprehensive experimental zones for big data, attempting to empower urban resource circulation and value-addition through data flows and facilitate upstream-downstream collaboration in the production chain, thereby seeking the green transformation of the urban industrial system. In terms of implementation effectiveness, Guizhou Province, which is the first batch of pilot zones, has ranked first in national digital economy development for six consecutive years and has successfully implemented the "One Cloud, One Network, One Platform" digital government services and the "Integration of Ten Thousand Enterprises" action, resulting in improvements in urban public basic services and optimization of industrial production operations, contributing to the formation of a high-quality governance pattern by the government."

The literature on the policy effects of big data comprehensive experimental zones mainly focuses on the assessment of economic effects and the evaluation of air pollution control [4]. On one hand, scholars have paid attention to the impact of big data on various sectors of the economy and society; this effort is a key element of the digital economy strategy. For example, big data can enhance the overall productivity of cities through information and communication technology [5] and can promote the improvement of both the quantity and quality of foreign direct investment [6]. Scholars have also observed the effects of big data on microlevel enterprises, such as its ability to promote industrial ecological transformation through technological innovation and energy consumption reduction [7]. Moreover, the role of big data in improving air quality and reducing haze and carbon emissions has been emphasized. Bing-Nan et al. [8] used the environmental Kuznets curve theory to demonstrate the significant effect of the digital economy in improving air quality, with emission reduction effects not only being effective locally but also contributing to a significant decrease in air pollution in neighboring cities. The research by Wang X and Zhong M [9] indicates that big data pilot policies can reduce smog and carbon emissions through industrial structure upgrading and the application of robots. Wei et al. [10] found that comprehensive experimental zones for big data can effectively reduce carbon emissions by promoting industrial upgrading, improving energy efficiency, and increasing the green overall factor productivity of the manufacturing industry. Compared to the analysis of the impact of policies in the big data comprehensive experimental zones, there are more studies analyzing the factors influencing pollution, such as government behavior [11], labor structure [12], industrial agglomeration [13], land finance [14], and economic growth goals [15, 16], and these factors are closely linked to environmental pollution emissions. Currently, most studies on the empowerment of big data in environmental pollution mainly focus on air pollution and often rely on single indicators to describe the environmental pollution variables, without fully considering the various types of pollutants emitted during urban industrial development. Moreover, relevant research only involves low-carbon governance in cities, with limited literature thoroughly examining the economic consequences of policy effectiveness in pollution control.

The role played by big data in achieving simultaneous improvements in economic quality and air quality within the framework of urban environmental governance cannot be ignored. Under the context of the "dual carbon" goal, can big data alleviate urban pollution and empower environmental governance? In what ways does big data influence pollution control in cities? The quality of urban environments is closely linked to the development of China’s new urbanization path. Can data empowerment in urban pollution control contribute to the formation of China’s new urbanization pattern? Clarifying the logical mechanism of these questions is of great practical significance for building a "Green China," promoting the development of the digital economy, and achieving sustainable economic development. The development of the urban environmental governance pattern requires more tools and methods. This article uses panel data from 283 prefecture-level cities from 2011 to 2019 to explore the impact of big data on urban environmental pollution control and examine the economic consequences of data empowerment in pollution control from the perspective of the urban‒rural dichotomy. The contributions of this article are as follows. First, the article empirically analyzes the impact of big data on urban environmental pollution control in China using the PSM-DID model, accounting for various pollutants emitted during urban industrial development, thus enriching the current research perspectives. Second, it incorporates public environmental concerns and green technological innovation into the analytical framework to further explore the implementation mechanisms of big data in empowering urban pollution control. Third, it further investigates the economic consequences of the policy effectiveness of big data in pollution control based on the perspective of the urban‒rural dichotomy and the introduction of new urbanization theory.

## Theoretical mechanisms

### How digital technology impacts urban environmental pollution

Digital technology pilot zones, which leverage emerging technologies such as 5G and cloud computing, can help manage urban pollution by integrating local industrial environments and other aspects. These zones can reduce energy consumption and waste of resources, as these are the primary sources of environmental pollutants. On the one hand, the internet has the ability to reduce the current energy storage costs and integrate these into the energy production and development process. For instance, big data projects such as the Energy internet can enhance energy efficiency while promoting the green transformation of the energy industry and reducing environmental pollution [17, 18]. Digital technology can help by reducing information asymmetry, empowering enterprises to search and collate information, and improving economic operation efficiency, thereby reducing the waste of resources [19, 20]. Regarding environmental pollution regulation, digital technology pilot zones can efficiently shape the green regulatory system using various technological applications, such as artificial intelligence, remote sensing technology, and digital technology platforms [21]. These tools can help city governments prevent and control environmental pollution and monitor environmental data such as water quality and carbon emissions in real time. This will significantly reduce urban environmental pollutant emissions and improve government environmental supervision and pollution control capabilities. Moreover, digital technology can enable the green transformation of cities by supporting new industries such as the sharing economy and platform economy. These industries can drive low-carbon and smooth economic operation by using digital networks to transform traditional production methods and provide technical support for renewable energy research and development. Smart manufacturing can lead to green processes and service innovation, further reducing local pollutant emissions [22]. As per Metcalfe’s Law, digital technology networks that are digitized, intelligent, and shareable will efficiently drive technological change and green transformation in pilot areas. This will empower city governments to manage the environment better and reduce local pollutant emissions. Based on this, we propose the following hypothesis:

Hypothesis 1: Digital technology can effectively reduce environmental pollutant emissions and empower urban environmental pollution management.

### Theoretical mechanisms of digital technology on urban environmental pollution

Given that digital technology has been a fundamental carrier for the development of the digital economy, the digital economy’s continued growth has in turn led to the proliferation and extension of digital technology [23, 24], playing an essential role in promoting urban environmental pollution management. Empowering urban subjects, digital technology can reduce urban environmental pollutant emissions by enhancing public environmental concern on the demand side and green technological innovation on the supply side.

First, digital technology can enhance public environmental concern, thereby reducing pollution emissions. The creation of digital technology pilot zones can improve residents’ lifestyles and enhance public awareness of green issues. With China’s rapid economic development, residents’ pursuit of a high quality of life has become increasingly urgent. New digital industries can stimulate public demand for green products and enhance residents’ concern for green and healthy living [25]. For instance, internet shopping platforms and environmental information websites play a crucial role in the construction of urban societies. By applying these new technologies, the public’s consumption channels can be expanded, shopping trips reduced, and a new way of living that is green and environmentally friendly encouraged. Thus, as digital technology networks advance, urban environmental pollution problems can be directly mitigated while forcing city governments to undertake environmental regulation initiatives to curb pollutant emissions. Moreover, digital technology-integrated pilot zones can bring together high-tech enterprises and high-quality human capital under local government technology subsidies and talent introduction policies [8]. Relevant studies have shown that high-end labor elements have higher-level requirements for urban commuting tools and living environments [26]. Driven by the pilot policy, both local residents and highly qualified foreign talent will have a higher concern for local environmental management, and thus will help reduce environmental pollution and promote the development of green and low-carbon urban transformation. Based on this, this paper proposes Hypothesis 2.

Hypothesis 2: Digital technology can reduce pollution emissions and empower urban environmental pollution management by enhancing public environmental concerns.

Second, digital technology can enhance green technology innovation, thereby reducing pollution emissions. Led by national-level integrated digital technology pilot zone policies, digital technology development can effectively breakdown spatial and temporal barriers, efficiently bring into play internet economies of scale and long-tail effects, and promote the spatial overflow of local knowledge and technology [27]. Being an important carrier of regional innovation activities, the efficient and shared flow of information technology can stimulate urban subjects to carry out R&D and innovation and cultivate new products, technologies, and services [28]. Unlike other innovation models, digital technology has green and low-carbon attributes [29]. IT-enabled innovation by microentities not only enhances the market’s competitive position by virtue of lower unit costs but also reduces the cost of environmental pollution control to achieve green and coordinated development [30]. From the perspective of the internal environment of enterprises, the integration of digital technology can lead to the intelligent transformation of enterprises, can optimize the current business management model, and achieve green and low-carbon development on the basis of forcing the research and development of clean technologies. From the perspective of the external environment, digital network construction is commonly implemented in pilot areas, and the advantages of internet integration can optimize resource allocation, efficiently improve production efficiency, and help upgrade the city’s industrial structure. With the transmission of favorable policy signals, high-tech enterprises and high-level technological achievements will gradually eliminate old, outdated "high energy consumption and high pollution" technologies and production models, leading the city to run smoothly in a green and sustainable direction. Based on this, this paper proposes Hypothesis 3.

Hypothesis 3: Digital technology can reduce pollution emissions and empower urban environmental pollution management by enhancing green technology innovation.

**Fig 1:**
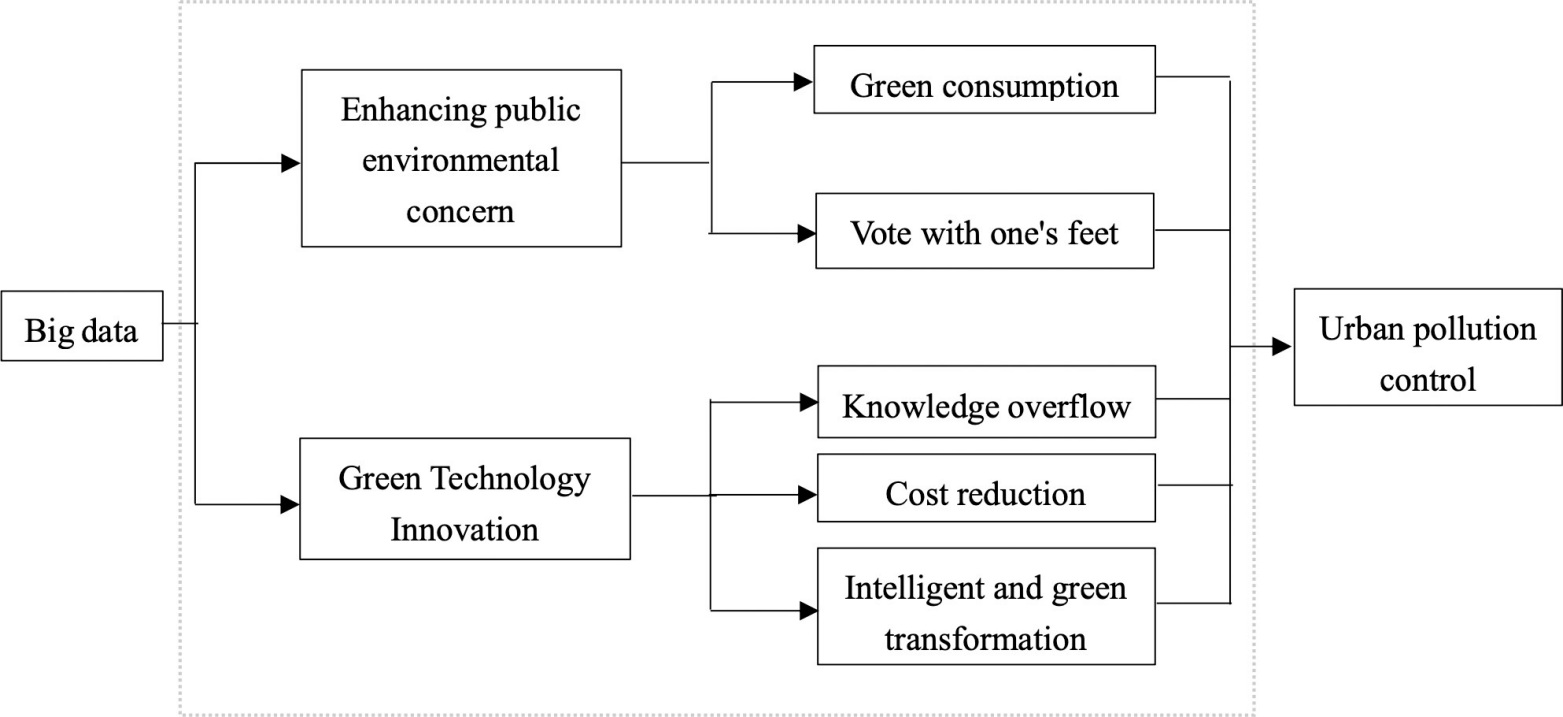
Logical framework of the paper.

Mechanism analysis frame diagram is shown in Fig 1.

## Material and methods

### Model set up

The objective of this paper is to investigate whether digital technology can reduce urban environmental pollution and improve urban environmental governance. To achieve this goal, the national-level integrated digital technology pilot areas are considered quasinatural experiments, with nonpilot areas as the control group and pilot areas as the treatment group. Since digital technology integrated pilot zones are not a single pilot policy, the econometric model in this paper is set up as shown below, drawing on the study of Beck et al. [31]

$${Pollution}_{it}=\alpha_{0}+\alpha_{1}Data+\alpha_{2}{Control}_{it}+\gamma_{i}+\mu_{t}+\varepsilon_{it}$$ (1)

The explanatory variable *Pollution*_*it*_ represents the regional environmental pollution level, while *Data* is the explanatory variable representing the national comprehensive experimental zone of digital technology. *Control*_*it*_ is the control variable, representing a series of variables affecting regional environmental pollution. The city fixed effect is represented by *γ*_*i*_, the year fixed effect by *μ*_*t*_, and the residual term by *ε*_*it*_, with *i* referring to the prefecture-level city and *t* to the year. In this paper, we focus on the coefficient *α*_1_ before *Data*. If *α*_1_ is significantly negative, it indicates that the national-level integrated digital technology pilot zone can significantly reduce environmental pollution in Chinese prefecture-level cities. In other words, digital technology can empower urban environmental pollution management.

### Definition of variables

The explanatory variable in this study is environmental pollution (***Pollution***_***it***_), which is mainly measured using the environmental pollution index. To measure urban environmental pollution, this paper adopts the entropy value method based on the research of Wang and Sun [32] The method involves two steps. First, considering the nonadditivity of industrial sulphur dioxide emissions, industrial wastewater emissions, and industrial smoke emissions, the "three wastes" are standardized and weighted using their respective coefficients. Second, the environmental pollution index of prefecture-level cities is obtained by a weighted summation. To ensure the accuracy of the findings, this paper draws on the study of Deng and Zhang [33], standardizing and logarithmizing the "three wastes" separately to portray urban environmental pollutants.

The core explanatory variable is the national digital technology comprehensive pilot zone (***Data***). In August 2015, the State Council issued the "Action Plan for Promoting the Development of Digital Technology", calling for "regional pilot projects". Guizhou province was the first pilot region, followed by nine provinces (municipalities directly under the Central Government), including Beijing and Tianjin, in the following year. Based on previous research on pilot policies [34], this paper adopts a multiperiod double-difference model. The explanatory variables in the baseline regression model are defined based on whether a different city i becomes a national digital technology pilot area in a different year t. If it becomes a pilot area in year t, then the core explanatory variable is 1 in that year and subsequent years; otherwise, it is 0.

Control variables: Population size (*Pop*): A larger population leads to increased resource consumption and deteriorating environmental quality [35]. This is measured by the logarithm of the total population at the end of the year. Fiscal autonomy (*Fd*): Higher fiscal autonomy allows local governments to have greater flexibility and resource mobilization capacity [36]. It is represented by the ratio of general budgetary revenue to general budgetary expenditure at the local level. Financial development (*Fin*): There is a close relationship between financial development and environmental pollution in China [37]. The level of financial development is measured by the ratio of the year-end balance of financial institution loans and deposits to regional GDP. Industrial structure (*Str*): The industrial structure is an important link between human activities and environmental quality, and changes in the industrial structure affect urban environmental pollution emissions [38]. It is represented by the ratio of the value added of the tertiary industry to the value added of the secondary industry. Internet development (*Fin*): The internet is an important channel for regulating resource allocation, and influencing pollution emissions, and governance. It has a significant impact on urban pollution [39]. It is measured by the logarithm of the number of broadband internet access users per hundred people. The descriptive statistics of the above variables are shown in Table 1.

**Table 1:** Descriptive statistics of variables.

Variable Type	Variable Name	Sample Size	Mean	Standard Deviation	Maximum	Minimum
Explained variables	*Pollution*	2547	0.164	0.139	0.005	0.693
Explanatory variables	*Data*	2547	0.130	0.336	0	1
Control variables	*Pop*	2547	5.878	0.680	4.013	7.647
	*Fd*	2547	0.465	0.221	0.102	0.999
	*Fin*	2547	0.986	0.565	0.315	3.358
	*Str*	2547	0.979	0.507	0.313	3.443
	*Inte*	2547	12.088	0.683	10.534	13.813

### Data sources

This paper uses a panel of 283 cities from 2011 to 2019, as the China Urban Statistical Yearbook 2021 does not report data on industrial smoke emissions and industrial wastewater emissions for each prefecture-level city in China in 2020. Public environmental concern data were obtained from internet searches, while green patent application data were obtained from the National Intellectual Property Database and matched with the “WIPO International Patent Classification Green List”. To address outliers, this paper performs bilateral Winsorize tail reduction on continuous variables and linear interpolation on missing data for some years.

## Results

### Parallel trend test

**Fig 2:**
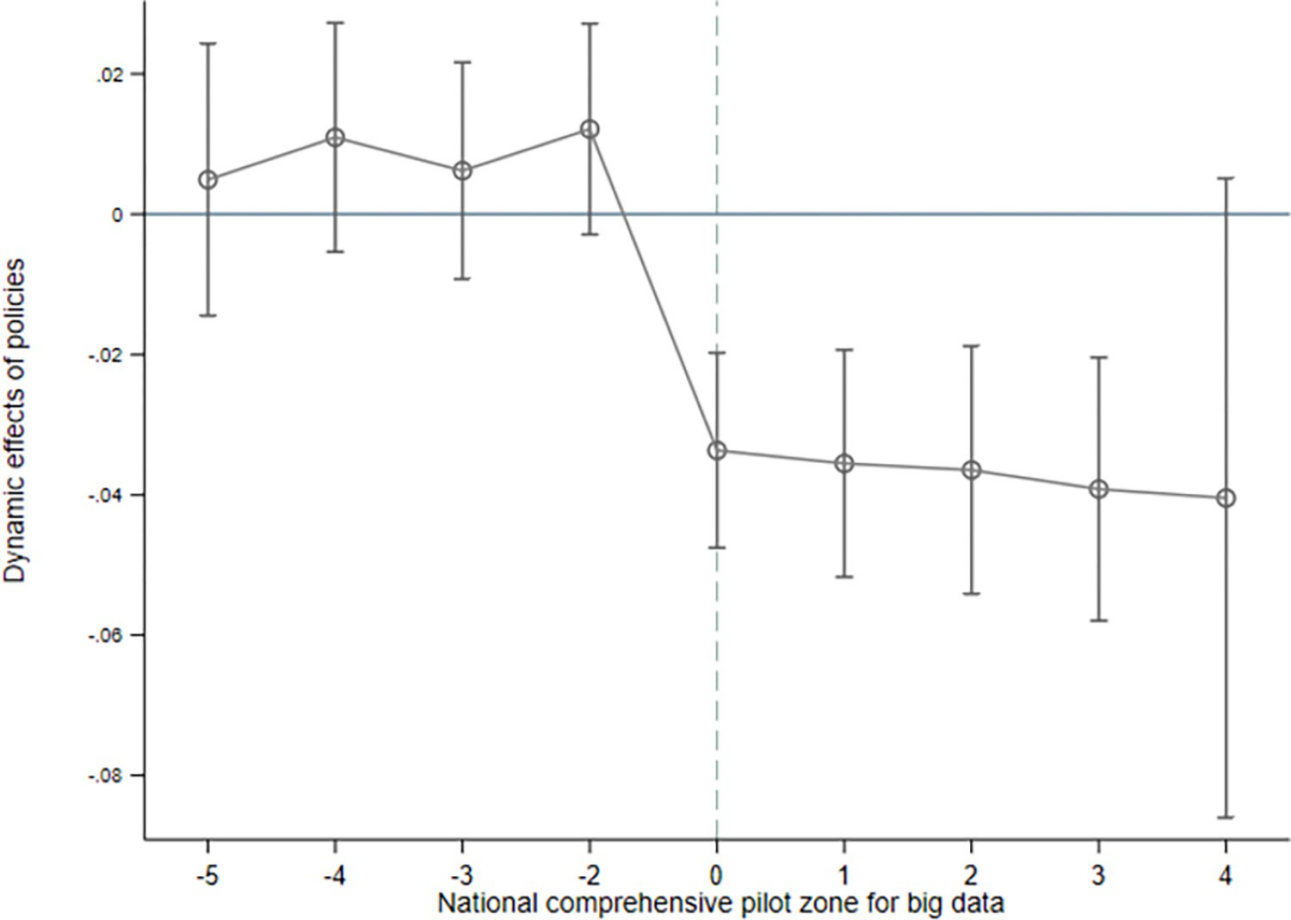
Parallel trend test results.

Before conducting the baseline regression analysis using the multiperiod double difference method, we first need to apply a parallel trend test. This test helps ensure that there are no significant differences between the control and treatment groups before the policy implementation and that there is a significant reversal between the two groups after the policy implementation. This is a necessary step to use the multiperiod double difference method. The specific model setting for the parallel trend test is as follows:

$${Pollution}_{it}=\beta_{0}+\beta_{1}\sum_{k=-5}^{k=4}{Data}_{k}(k\neq -1)+\beta_{2}{Control}_{it}+\gamma_{i}+\mu_{t}+\varepsilon_{it}$$ (2)

where *k* denotes the period before and after the policy implementation. In this paper, we use the year before the implementation of the national-level digital technology comprehensive experimental zone as the base period. As shown in Fig 2, there is a dynamic effect of the national-level comprehensive pilot zone for digital technology on environmental pollution in prefecture-level cities. The regression coefficients for the pilot zone were not significant before the policy was implemented, and there was no significant upward trend. After the policy was implemented, the pilot zone had a significant inhibitory effect on environmental pollution in the prefecture-level cities at the time of policy implementation, and there was a long-term dynamic process. The parallel trend test is thus passed, meeting the important prerequisite of using a multiperiod double difference model.

### Baseline regression analysis

The benchmark regression results of this study are shown in Table 2. Columns (1) to (2) indicate that regardless of whether control variables are included, there is a significant negative correlation between national-level big data comprehensive pilot zones and urban environmental pollution at the 1% significance level, indicating that big data can indeed empower urban environmental pollution governance. Considering the inseparable relationship between urban economic development and industry, to ensure the accuracy of the research results, this study further conducts regression analysis on the "three wastes" at this point. Columns (3) to (5) show that the regression coefficients for national-level big data comprehensive pilot zones are -0.281, -0.063, and -0.179, respectively, indicating that the implementation of pilot policies can significantly reduce industrial sulfur dioxide, wastewater, and smoke dust emissions by 28.1%, 6.3%, and 17.9%, respectively.

**Table 2:** Baseline regression results on environmental pollution for national-level integrated digital technology test areas.

Variables	Baseline regression		Industrial sulphur dioxide	Industrial wastewater	Industrial soot
	(1)	(2)	(3)	(4)	(5)
	*Pollution*	*Pollution*	*Lnso*2	*Lnwater*	*Lnsmoke*
*Data*	-0.045*** (-8.556)	-0.043*** (-7.835)	-0.281*** (-6.685)	-0.063** (-2.016)	-0.179*** (-3.868)
Control variables	No	Yes	Yes	Yes	Yes
Year fixed	Yes	Yes	Yes	Yes	Yes
City fixed	Yes	Yes	Yes	Yes	Yes
Constant term	0.170*** (132.180)	-0.066 (-0.277)	-2.876 (-1.128)	7.071*** (6.348)	-2.171 (-1.087)
Sample size	2547	2547	2547	2532	2529
R^2^	0.859	0.861	0.870	0.912	0.836

Note: *, **, ***, denote passing the significance test at 10%, 5% and 1%, respectively. Control variables include population size, fiscal autonomy, financial development, industrial structure and internet development, with standard errors in brackets. Same as below.

This conclusion is consistent with related studies. Based on the G20 countries and nonlinear models, Nguyen et al. [40] and Lahouel et al. [41] found that the application of information and communication technology not only promotes regional economic development but also reduces carbon emissions, facilitating green transformation and development. From the perspective of governance, the research many scholars indicates that the application of big data can indeed improve the efficiency of government environmental regulation. Cloud computing, remote sensing technology, and other tools enable regional governments to achieve real-time dynamic monitoring of environmental pollution, air quality, and resource data [21].

Specifically, big data have a stronger inhibitory effect on industrial sulfur dioxide emissions in Chinese cities. A possible reason for this is that sulfur dioxide is an important indicator of industrial pollution in Chinese cities. With its unique attributes, such as green innovation, big data can regulate and control industrial emissions through clean production technologies, thereby reducing the burden of urban pollution.

### Analysis of heterogeneity

National-level big data comprehensive pilot zones do have a significant inhibitory effect on urban environmental pollution, but different types of cities may have different policy effects. Given the perspective of this study’s research theme, considering the close correlation between regional economic conditions, digital infrastructure, and the intensity of policy radiation, as well as the close relationship between energy efficiency, clean structure, and environmental emissions, we focus on examining whether these three aspects will have different impacts on the effectiveness of big data in pollution control.

One test is based on whether the city is an old industrial base. Rapid industrial development often comes at the expense of resources in exchange for the environment. Old industrial bases are in the northeastern part of the country and have a wide range of industrial sectors and high concentration. As a result, environmental pollution problems in old industrial bases may be more serious than in nonold industrial bases [42]. In this study, we divided the urban sample into nonold industrial bases and old industrial bases and separately investigated the effect of the Digital Technology Comprehensive Pilot Zone policy on these two types of areas. The results in columns (1) and (2) of Table 3 show that the national-level integrated pilot zones for digital technology have a more significant inhibitory effect on pollutant emissions in old industrial bases but do not have a significant effect on environmental pollution in nonold industrial bases. A possible reason for this is that old industrial bases refer to the early traditional industrial regions that were formed during China’s planned economy era, and these are all located at transportation hubs in China. Therefore, compared to other regions, these areas have a solid industrial foundation and possess strong technological accumulation.

**Table 3:** Results of the heterogeneity analysis of environmental pollution in the national-level integrated digital technology test area.

Variables	Nonold industrial base	Old industrial base	Low numerical base areas	High numerical base areas	Low energy efficiency areas	High energy efficiency areas
	(1)	(2)	(3)	(4)	(5)	(6)
	*Pollution*	*Pollution*	*Pollution*	*Pollution*	*Pollution*	*Pollution*
*Data*	-0.007 (-1.212)	-0.073*** (-8.230)	-0.037*** (-5.756)	-0.059*** (-4.760)	-0.053*** (-6.307)	-0.027*** (-3.433)
Control variables	Yes	Yes	Yes	Yes	Yes	Yes
Year fixed	Yes	Yes	Yes	Yes	Yes	Yes
City fixed	Yes	Yes	Yes	Yes	Yes	Yes
Difference between groups	0.066		0.022		-0.026	
Empirical P value	0.000		0.000		0.002	
Constant term	-1.847*** (-5.146)	1.203*** (2.988)	-0.059 (-0.167)	-1.501* (-1.940)	-0.355 (-1.175)	-1.799*** (-3.202)
Sample size	1485	1044	1751	738	1172	1327
R^2^	0.870	0.868	0.865	0.884	0.883	0.880

Another test is based on the level of development of the city’s digital base. There are some differences in digital infrastructure across China. High digital base areas are more conducive to the pilot policy in terms of playing its role in optimizing the allocation of resources and the long-tail effect of the network to help control urban environmental pollution compared to low digital base areas. This study divides the urban sample into low-digital base areas and high-digital base areas, using the mean value of the digital base as the boundary. The results in columns (3) and (4) of Table 3 show that national-level integrated digital technology pilot zones have a significant inhibiting effect on environmental pollution in both high and low digital base areas. However, the empirical p-value between the two groups is zero, indicating that the integrated digital technology pilot zones have a stronger effect on environmental pollution control in cities with a high digital base. Based on the spatial distribution in China, it is evident that areas with a high level of digital infrastructure are typically found in economically developed regions that are equipped with well-established network systems and data frameworks. Digitalization in these regions has a more significant impact on the economy and society compared to other areas. Therefore, with the support of the big data experimental zone, the production value chain in these regions will achieve a green and clean transformation.

A third test is based on the level of urban energy efficiency. Energy use efficiency is closely related to urban environmental pollution management. Environmental pollution is a more serious problem in areas with lower energy efficiency. Digital technology can promote the green transformation of the economy and empower local pollution management through data flow from various aspects, such as technology and capital. This study divides the urban sample into low-energy efficiency regions and high-energy efficiency regions, using the mean energy efficiency value as the boundary. Energy efficiency is measured as the logarithm of the ratio of regional GDP to energy consumption [43]. The results in columns (5) and (6) of Table 3 show that the national-level integrated pilot areas for digital technology have a more pronounced suppressive effect on the emissions of environmental pollutants in low-energy-efficiency regions. A possible reason for this is that, unlike low-energy regions, high-energy regions have greener and cleaner energy structures in their businesses, more comprehensive and efficient network regulatory systems, and a higher overall degree of industrial green transformation. Therefore, the impact of big data support on government pollution control is relatively lower in these regions. On the other hand, regions with lower energy efficiency have a relatively backward industrial foundation, and the empowerment of big data will prompt these areas to quickly transition to clean and green technologies. Combining information technology applications in industrial green practices and government regulations will contribute to the establishment of local environmental pollution control systems.

### Robustness tests

#### PSM-DID

**Fig 3:**
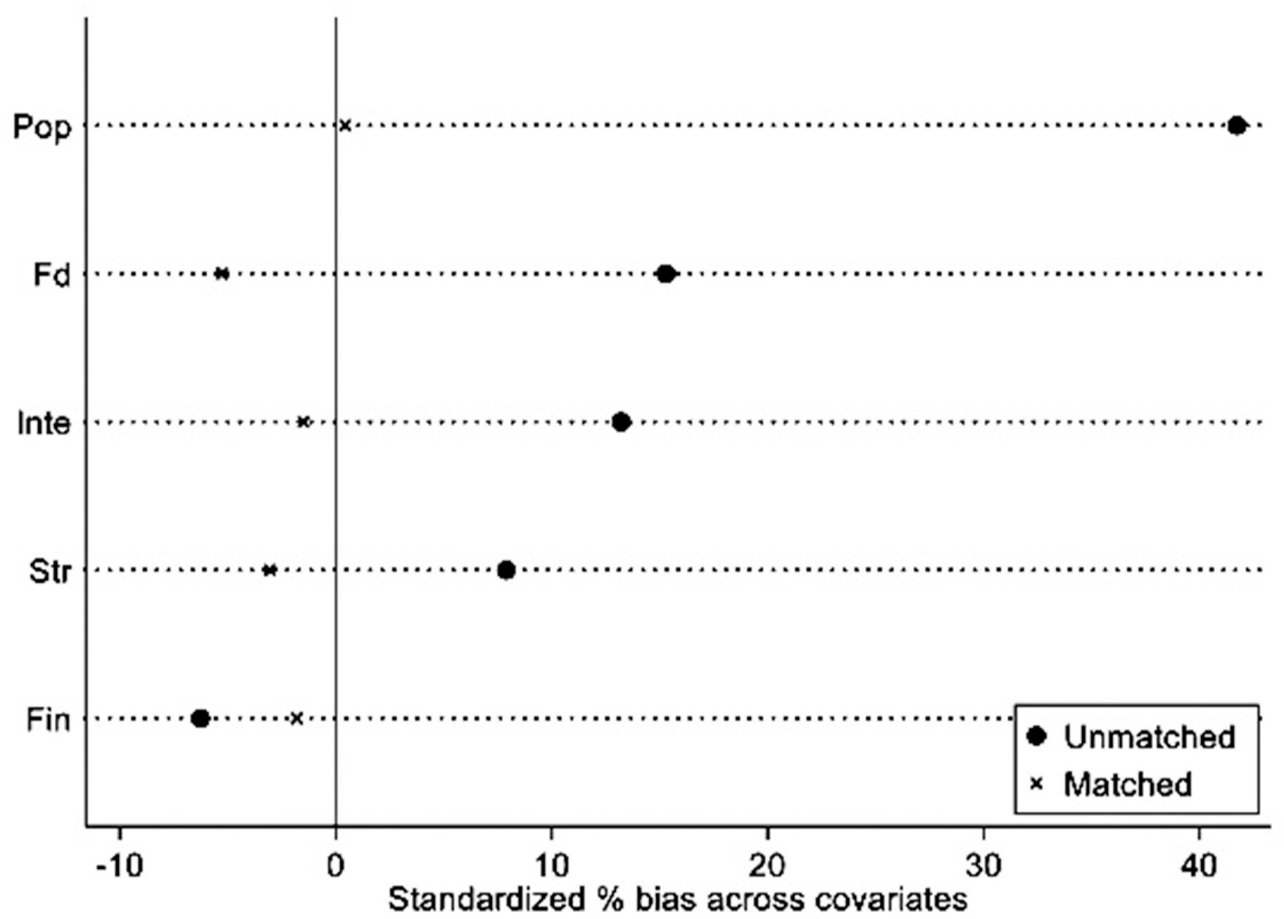
Balancing test of propensity score matching (PSM).

To ensure the accuracy of the baseline regression results, this study continues to employ the propensity score matching with difference-in-differences (PSM-DID) method. Specifically, nearest neighbor matching is primarily used in this study to match city samples on an annual basis. (We also use radius matching and Mahalanobis metric matching, and it is found that the results are still robust and reliable. Considering that the nearest neighbor matching is the most commonly used matching method in PSM, only its results are shown here.) Nearest neighbor matching matches the treated group research subjects and makes full use of information from the treated group, and this is commonly used in empirical research. According to "The average effect of PSM," the Average Treatment Effect on the Treated (ATT) value is 2.72, and thus it is statistically significant at the 1% level. This indicates a causal relationship between the core explanatory variable and the dependent variable. As shown in Fig 3, the standardized biases of all covariates are less than 10% and are significantly smaller than the standardized biases before matching. After removing samples from the nonoverlapping region, this study performs a new regression on the matched sample size. The regression results in Table 4, Column (1), after conducting annual PSM-DID, demonstrate that the regression coefficient of the national-level big data comprehensive experimental zone remains significantly positive at the 1% statistical level. The coefficient size is comparable to the baseline regression results, and the research conclusion of this study remains unchanged.

**Table 4:** Results of the robustness of the national-level integrated digital technology test area to environmental pollution.

Variables	PSM-DID	Exclude low carbon cities	Exclude broadband China policy	Control for omitted variables	Control for provinces and clustering	Top and bottom 5% tail reduction
	(1)	(2)	(3)	(4)	(5)	(6)
	*Pollution*	*Pollution*	*Pollution*	*Pollution*	*Pollution*	*Pollution*
*Data*	-0.040*** (-7.232)	-0.078*** (-10.072)	-0.016*** (-2.643)	-0.036*** (-6.344)	-0.038*** (-2.920)	-0.031*** (-6.473)
Missing variables	No	No	No	Yes	No	No
Control variables	Yes	Yes	Yes	Yes	Yes	Yes
Year fixed	Yes	Yes	Yes	Yes	Yes	Yes
City fixed	Yes	Yes	Yes	Yes	Yes	Yes
Constant term	-0.015 (-0.059)	-1.212*** (-3.056)	-1.230*** (-3.219)	-0.890*** (-2.707)	-0.948* (-1.656)	-0.214 (-0.578)
Sample size	2489	1400	1523	2349	2409	2409
R^2^	0.859	0.858	0.858	0.866	0.866	0.863

### Excluding policy interference

To avoid bias caused by other regional economic pilot policies in China, this study chooses to exclude low-carbon city construction and broadband China policies from the low-carbon and network perspectives. Low-carbon city construction is closely related to urban environmental pollution control, while broadband China has a crucial role to play in digital technology development and industrial green transformation. As shown in columns (2) and (3) of Table 4, when the relevant policy interference is excluded, the national-level integrated digital technology pilot zones can still significantly reduce urban environmental pollutant emissions and empower urban environmental pollution management.

### Placebo test

**Fig 4:**
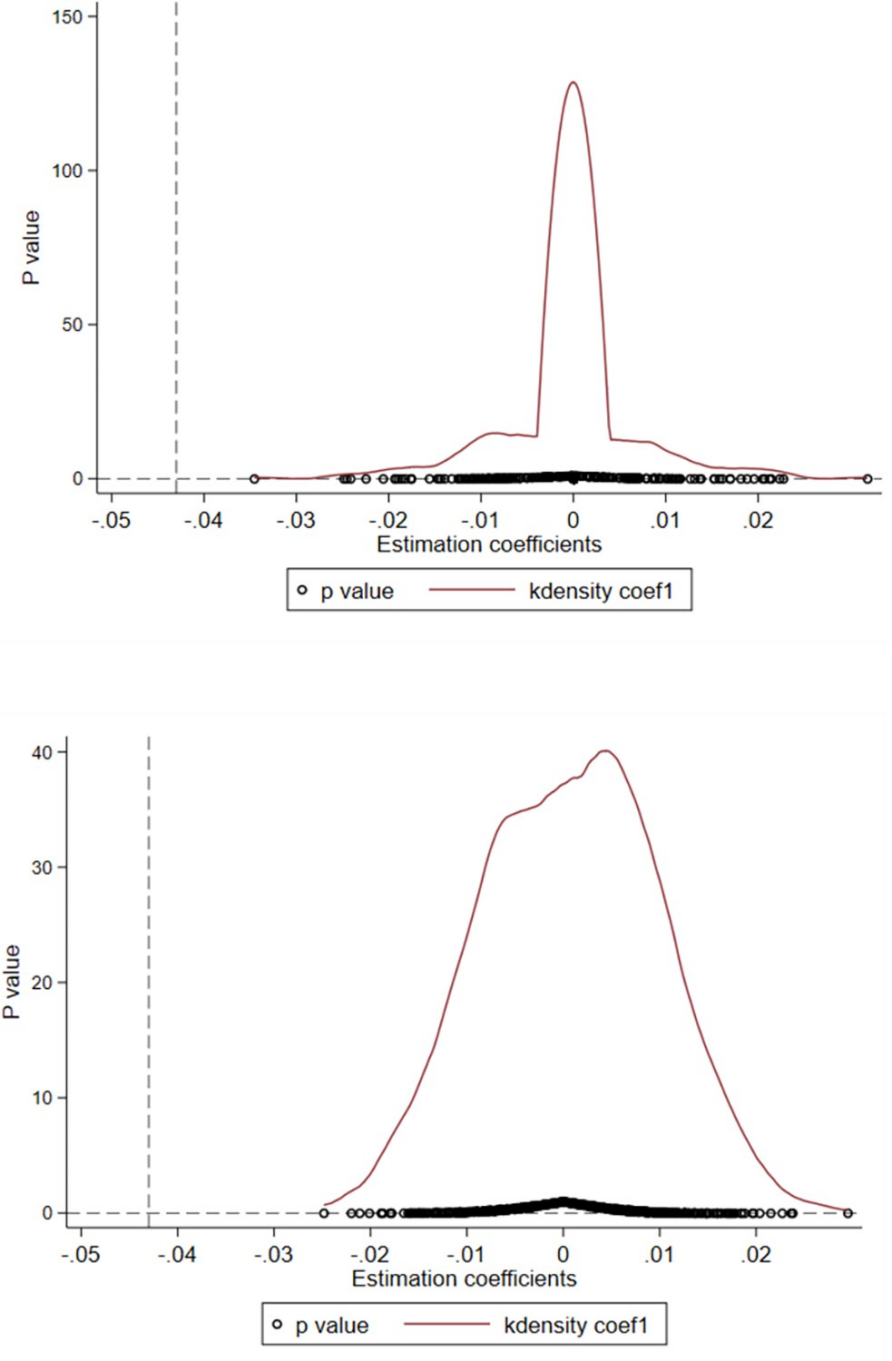


To address the influence of unobservable variables on the baseline regression results, this study reselects the pilot cities and pilot years by random sampling. The core explanatory variables are reconstructed based on the results of random sampling and brought into the baseline model regression. After repeating the above operation 200 times and 500 times, a placebo test was conducted in this study. As shown in Fig 4, the simulated values distribution of the explanatory variables reconstructed regressions are mostly concentrated at approximately 0, and the p-value is greater than 0.1. The true regression coefficient of this study (-0.043) is significantly more abnormal than the simulated values of the regressions in the placebo test, indicating the robust reliability of the findings from the counterfactual perspective.

### Other robustness tests

To further ensure the robustness of the findings, this study takes the following measures: controlling for omitted variables by including the share of science and technology expenditure and the degree of external linkages as additional explanatory variables. These variables are measured by the ratio of science and technology expenditure to local fiscal general budget expenditure, the amount of actual foreign investment utilized, and the ratio of GDP, respectively. Adding province fixed effects and clustering standard errors, and applying a 5% upper and lower tailing, Columns (4) to (6) of Table 4 show the results of the robustness tests described above. The conclusion that the national-level integrated digital technology pilot zone can significantly reduce urban environmental pollution emissions remains unchanged, as indicated by the significance and sign of the regression coefficients.

## Mechanism testing and further discussion

### Mechanism analysis

The theoretical mechanism suggests that the national-level digital technology comprehensive experimental zone can enhance public environmental concern and green technology innovation by bringing together talent and technology. This can reduce local environmental pollutant emissions and help the construction of urban environmental pollution control. To explore the mechanism and channels of digital technology empowerment, this study constructed the following model.


$${Pollution}_{it}=\delta_{0}+\delta_{1}Data+\delta_{2}{Metvar}_{it}+\delta_{3}Data\times {Metvar}_{it}+\delta_{4}{Control}_{it}+\gamma_{i}+\mu_{t}+\varepsilon_{it}$$ (3)

Among the variables, *Metvar*_*it*_ is the mechanism variable in this article, and it is used to characterize environmental concern and green technological innovation. There is relatively more research on the relationship between environmental concern, green technological innovation, and environmental pollution. This article focuses on whether the comprehensive demonstration zone of big data can have a promoting effect on the mechanism variable, thus further empowering environmental pollution control.

The mechanism of public environmental awareness. The mechanism of public environmental awareness plays a role in stimulating the aggregation of high-quality talents while facilitating the transformation and upgrading of industrial structure through the phased implementation of pilot policies. Highly skilled human capital, which tends to pursue a higher quality of living environment, continuously influences and enhances local public attention towards the environment. Drawing on the research conducted by Wu Libo et al. (2022) [44], this study primarily employs the Baidu search index to measure public environmental awareness. Specifically, the study initially selects the commonly concerned keyword "environmental pollution" among urban residents, taking into account "haze" as a pollution phenomenon directly perceived by the public and includes it as a keyword. Subsequently, the study conducts searches for the aforementioned environmental keywords separately on PC and mobile platforms, aggregates and logarithmically transforms the search indices, resulting in the mechanism variables of this study, namely public environmental awareness (*Env*) and public pollution awareness (*Smo*). Table 5 presents the mechanism regression results of Model (3) in columns (1) and (2). The regression coefficients indicate that both environmental awareness and haze awareness have significant inhibitory effects on the emission of pollutants in the prefecture-level cities of the comprehensive big data experimental zones. The《2007 National Survey on Work Environment Awareness》has already shown that environmental pollution has become the fourth major concern for the Chinese public. With the advent of the era of technological innovation leading to high-quality development, the domestic public’s emphasis on the quality of residential life is increasing, compelling local governments to adopt effective environmental protection measures. Big data, capable of empowering technological iteration and advancement, plays a crucial role in urban environmental governance, such as the implementation of intelligent water management systems for river joint prevention and control.

**Table 5:** Results of the mechanism test of the national-level comprehensive test area of digital technology on environmental pollution.

Variables	Public environmental concern	Public haze concern	Green technology innovation	Green innovation quality	Green innovation quantity
	(1)	(2)	(3)	(4)	(5)
	*Env*	*Smo*	*Gin*	*Inv*	*Ninv*
***Data***	0.106*** **(3.929)**	0.128*** **(4.523)**	0.603*** **(3.624)**	0.277*** **(3.127)**	0.326*** **(3.819)**
**Control variables**	**Yes**	**Yes**	**Yes**	**Yes**	**Yes**
**Year fixed**	**Yes**	**Yes**	**Yes**	**Yes**	**Yes**
**City fixed**	**Yes**	**Yes**	**Yes**	**Yes**	**Yes**
**Constant term**	**-2.221** **(-1.606)**	**-0.515** **(-0.354)**	-42.751*** **(-3.985)**	-27.128*** **(-4.819)**	-15.623*** **(-2.950)**
**Sample size**	**2485**	**2462**	**2519**	**2519**	**2519**
**R** ^**2**^	**0.918**	**0.965**	**0.825**	**0.818**	**0.808**

The mechanism of green technological innovation. National-level comprehensive big data experimental zones can achieve full coverage of green technology in the production value chain through new infrastructures such as the Internet of Things and cloud computing, thereby reducing urban environmental pollution. This article primarily explores the impact of green technological innovation on empowering urban environmental pollution control through big data. Considering the uniqueness of invention patents among the three types of patents, this article further divides green technological innovation from the perspectives of "quantity" and "quality," drawing from the classification method proposed by Song et al.) [45]. Specifically, green technological innovation is measured by the number of green patents per 10,000 people (*Gin*), the number of green invention patents per 10,000 people to measure the quality of green innovation (*Inv*), and the number of green utility model patents per 10,000 people to measure the quantity of green innovation (*Ninv*). Columns (3) to (5) in Table 5 present the results of the mechanism regression for green technological innovation. The regression coefficients between national-level comprehensive big data experimental zones and green technological innovation, quality of green innovation, and quantity of green innovation all show a significant negative correlation at the 1% level of statistical significance. This indicates that the empowerment of big data can indeed significantly enhance the level of green innovation in the pilot areas, alleviate local environmental pollution problems, and contribute to urban pollution control efforts. This conclusion reaffirms the research by Anderson [46], stating that technological innovation can indeed reduce regional environmental pollution. At the same time, whether it is noncore content innovation or core technological innovation [47], big data can utilize information technology for integrated development, thereby reducing the emissions of the "three wastes" in urban industries.

### Further analysis

Research conducted by scholars both at home and abroad has shown that the digital dividend can help integrate urban and rural commerce, increase employment rates and promote new urbanization [48, 49]. Considering the core concepts of industrial transformation, green and low-carbon development, and a livable environment emphasized by the new urbanization and guided by the scientific development concept, there is a certain correlation with regional pollution control. This paper focuses on how digital technology can amplify the role of environmental pollution management in advancing China’s new urbanization pattern, as this is guided by the scientific outlook on development, focusing on industrial transformation, green and low-carbon, and environmental livability. Using Xuan Ye and Peng Jie’s study, this paper analyses the economic consequences of digital technology-enabled urban environmental pollution management. New urbanization variables are portrayed in four dimensions: demographic, economic, social, and environmental. The paper calculates new urbanization indicators based on the entropy value method and adopts principal component analysis to ensure research accuracy. The specific model is constructed as follows [50]:

$${Ura}_{it}=\varphi_{0}+\varphi_{1}{Pollution}_{it}+\varphi_{2}{Control}_{it}+\gamma_{i}+\mu_{t}+\varepsilon_{it}$$ (4)

$${Ura}_{it}=\omega_{0}+\omega_{1}{Pollution}_{it}+\omega_{2}{Pollution}_{it}\times Data+Data+\omega_{3}{Control}_{it}+\gamma_{i}+\mu_{t}+\varepsilon_{it}$$ (5)

*Ura*_*it*_ represents new urbanization. Model (4) is used to verify the relationship between urban environmental pollution and new urbanization, with *ω*_2_ representing the coefficient of the interaction term in model (5). If environmental pollution inhibits new urbanization and *ω*_2_ is significantly positive, this suggests that digital technology can indeed amplify the effect of environmental pollution control and promote the formation of a new urbanization patterns in China.

Table 6 presents the regression results for further analysis, with column (1) showing the baseline finding that national-level digital technology integrated pilot zones can reduce urban environmental pollutant emissions and help with pollution control. Columns (2) and (4) in Table 6 are the regression results of model (4), indicating that environmental pollution has a significant hindering effect on the new urbanization process. By verifying model (5), this paper yields columns (3) and (5) in Table 6, showing that the interaction term between the national-level integrated digital technology pilot zone and environmental pollution remains significantly positively correlated at a statistical level of 1%, indicating that digital technology-enabled urban pollution management can further promote the formation of a new urbanization pattern in China. Based on the facts, it can be observed that unlike traditional urbanization, the new type of urbanization guided by the scientific development concept aims to prioritize people and emphasizes key aspects such as coordinating urban and rural development, industrial transformation, efficiency, low carbon emissions, resource efficiency, livable environment, and social harmony. The goal is to enhance the sense of gain and happiness of both urban and rural residents. Therefore, the new type of urbanization is closely related to regional environmental quality. Big data, with communication technology at its core, can drive urban industrial transformation and green development. For instance, the development of artificial intelligence can optimize urban public services, and big data monitoring platforms can regulate pollution emissions. The research and development of these emerging digital technologies continuously change residents’ lifestyle concepts and the production and transportation processes of clean products. They efficiently contribute to pollution control in cities and promote low carbon practices in urban and rural areas, thereby accelerating the formation of China’s new urbanization pattern.

**Table 6:** Results of the further analysis of environmental pollution in the national-level integrated digital technology test area.

Variables	Baseline Regression	New Urbanization (Entropy Method)		New Urbanization (Principal Component Analysis)	
	(1)	(2)	(3)	(4)	(5)
	*Pollution*	*Urb*	*Urb*	*Urb*	*Urb*
*Data*	-0.043*** (-7.835)		-0.006*** (-4.681)		-0.070*** (-3.645)
*Pollution*		-0.024*** (-4.218)	-0.022*** (-3.999)	-0.149** (-2.322)	-0.130** (-2.032)
*Data*×*Pollution*			0.052*** (5.734)		0.540*** (4.462)
Control variables	Yes	Yes	Yes	Yes	Yes
Year fixed	Yes	Yes	Yes	Yes	Yes
City fixed	Yes	Yes	Yes	Yes	Yes
Constant term	-0.066 (-0.277)	-0.060 (-0.736)	-0.062 (-0.749)	-2.720*** (-3.197)	-2.710*** (-3.178)
Sample size	2547	2376	2376	2376	2376
R^2^	0.861	0.983	0.983	0.977	0.977

## Conclusion and policy recommendations

China is undergoing a digital revolution, and it is crucial to achieve sustainable economic development to comply with the new technological revolution and achieve high-quality development. This paper explores the environmental pollution control effects of China’s cities from the perspective of digital technology and examines the close link between digital technology-enabled pollution control and new urbanization. Using data from 283 prefecture-level cities in China from 2011 to 2019 for empirical testing, the following conclusions were drawn: (1) National-level digital technology comprehensive pilot zones can significantly reduce environmental pollutant emissions and empower urban environmental pollution control construction in Chinese cities; the findings are proven upon conducting robustness tests such as the parallel trend test, PSM-DID method, and placebo test. (2) The inhibitory effect of digital technology empowerment on environmental pollution in old industrial bases, high digital bases, and low energy efficiency areas is more pronounced than in nonold industrial bases, low digital bases, and high energy efficiency areas. (3) National-level digital technology integrated pilot zones can help urban environmental pollution control by enhancing public environmental concern and green technological innovation, while digital technology-enabled pollution control construction is conducive to accelerating the formation of a new urbanization pattern in China.

Based on the above research findings, the following policy recommendations are proposed: (1) cultivate core digital competitiveness and stimulate new momentum for green transformation; and (2) increase "new infrastructure" facilities, enhance the breadth and depth of digital coverage, and promote the integration of digitalization and green development. Digital technology platforms and the long-tail effect of the internet are used to breakdown barriers to the diffusion of resource elements, help the low-carbon transformation of urban industries, and realize the deep and coordinated development of industries and cities. (3) Optimize the spatial layout of digital China and explore new paths for green development. We should account for the differences in urban characteristics and reasonably carry out top-level design and development planning. Accelerate the efficient integration and circular value-added of data and information in nonold industrial bases and other areas and promote the sharing of resources up and down the industrial chain and the renewal and iteration of production lines. It is important to continuously improve the carrying capacity of urban resources and digital development and optimize the efficiency of local green economic development while enhancing public awareness of environmental protection and helping build urban environmental pollution control. (4) Promote the integration and development of digitalization and urbanization and establish a new image of green transformation. The construction of digital technology and information should be relied upon to achieve high-quality development in many aspects of urban and rural employment, industry and social ecology so that urban and rural areas can share the welfare effects of digital empowerment. Digital platforms can be used to cultivate a high-quality workforce and high-level network supervision facilities, clean production value chains while enhancing the efficiency of government environmental regulation, and achieve the goal of new urbanization with a low-carbon and livable environment and green coordination between urban and rural areas.

This article has the following limitations. First, considering that the data on industrial wastewater and industrial smoke emissions for 2020 were not published in the "China Urban Statistical Yearbook," this article only retains data from 2011 to 2019 for calculating the environmental pollution index. In future research, it would be beneficial to add the data for 2020 and ensure its timeliness to dynamically examine the current state of big data empowering China’s urban pollution control. Second, the mechanism examination in this article mainly focuses on the perspectives of the public and enterprises, exploring the roles of environmental awareness and green innovation in mitigating environmental pollution through big data. In future research, it would be worthwhile to further investigate the influence of government environmental goals and constraints on the effectiveness of pollution control through big data. This aspect represents a crucial component of urban environmental management.

## Supporting information

S1 File (ZIP)
