# Correction: Can big data reduce urban environmental pollution? Evidence from China’s digital technology experimental zone

**DOI:** 10.1371/journal.pone.0317020

**Published:** 2025-01-02

**Authors:** Jiahao Zhang, Fusheng Liang, Peng Gao

There are errors in the author affiliations. The correct affiliations are as follows:

Jiahao Zhang^1^, Fusheng Liang^2^, Peng Gao^3^

**1** College of Economics and Management, Shanghai Ocean University, Shanghai, China, **2** Institute of Food and Strategic Reserves, Nanjing University of Finance and Economics, Nanjing, China, **3** College of Economics and Management, South China Agricultural University, Guangzhou, China.
